# SHNN-CAD^+^: An Improvement on SHNN-CAD for Adaptive Online Trajectory Anomaly Detection

**DOI:** 10.3390/s19010084

**Published:** 2018-12-27

**Authors:** Yuejun Guo, Anton Bardera

**Affiliations:** Graphics and Imaging Lab, Universitat de Girona, Campus Montilivi, 17071 Girona, Spain; yuejun.guo@udg.edu

**Keywords:** online anomaly detection, trajectory data, adaptive anomaly threshold, Hausdorff distance with constraint window

## Abstract

To perform anomaly detection for trajectory data, we study the Sequential Hausdorff Nearest-Neighbor Conformal Anomaly Detector (SHNN-CAD) approach, and propose an enhanced version called SHNN-CAD${}^{+}$. SHNN-CAD was introduced based on the theory of conformal prediction dealing with the problem of online detection. Unlike most related approaches requiring several not intuitive parameters, SHNN-CAD has the advantage of being parameter-light which enables the easy reproduction of experiments. We propose to adaptively determine the anomaly threshold during the online detection procedure instead of predefining it without any prior knowledge, which makes the algorithm more usable in practical applications. We present a modified Hausdorff distance measure that takes into account the direction difference and also reduces the computational complexity. In addition, the anomaly detection is more flexible and accurate via a re-do strategy. Extensive experiments on both real-world and synthetic data show that SHNN-CAD${}^{+}$ outperforms SHNN-CAD with regard to accuracy and running time.

## 1. Introduction

Thanks to advanced location-aware sensors, massive trajectory data are generated every day, which requires effective information processing techniques. The main objective of anomaly detection is to pick out anomalous data which are significantly different from the patterns that frequently occur in data. A lot of applications benefit from automatic anomaly detection of trajectory data, such as video surveillance [1,2,3], airspace monitoring [4], landfall forecasts [5], and so on.

A variety of approaches have been proposed for the task of trajectory anomaly detection [6], however, most of them have limitations of computational cost or parameter selection [7], leading to the difficulty in reproducing experimental results. Usually, the trajectory of a moving object is collected and stored as a sequence of sample points which record the location and timestamp information, but the complementary information of data is lacking. Here, the complementary information refers to information about the number of patterns included, the trajectories labeled with certain patterns, the abnormal pattern, and so on, which can help the analysis of data. To automatically find this information, unsupervised approaches are commonly applied. In this case, it is not straightforward or easy to finely tune the parameters for these approaches to cope with different kinds of data. Although some approaches make effort to estimate the parameters simply by experience and a lot of experiments or complicatedly by introducing more assisted parameters and rules, the parameter setting is not trivial with respect to different distributions. Especially, for online handling of massive datasets, the low computational complexity is of great importance. Thus, it is better to avoid complex and time-consuming pre-processing on data.

Based on the theory of conformal prediction, Laxhammar et al. successively proposed its application in anomaly detection [8], and then the Similarity based Nearest Neighbour Conformal Anomaly Detector (SNN-CAD) [9] for online learning and automatic anomaly detection, and further introduced a relatively complete algorithm called Sequential Hausdorff Nearest-Neighbor Conformal Anomaly Detector (SHNN-CAD) [10] by improving the description and details of their previous works with more comprehensive discussion of previous works and explanation of the algorithm. SHNN-CAD has the following main advantages. First, it deals with raw data, which prevents the problems of information loss from dimension reduction, and over-fitting from modeling. Second, it makes use of the direct Hausdorff distance to calculate the similarity between trajectories. As is well known, selecting a proper distance measure is still a challenge when the trajectories in a set have unequal lengths (trajectory length refers to the number of sample points) due to sampling rate, sampling duration, and moving speed. To solve this issue, the direct Hausdorff distance is a good choice as it is parameter-free, and it is able to handle the case of unequal length. Third, SHNN-CAD is parameter-light and makes no assumption on data structure. The authors provided a method to adjust the anomaly threshold based on the desired alarm rate or the expected frequency of anomalies. Fourth, SHNN-CAD can perform online anomaly detection, which enables the increase of data size.

In this paper, we propose SHNN-CAD${}^{+}$ to enhance the performance of SHNN-CAD. Compared with the previous approach, SHNN-CAD${}^{+}$ has the following improvements:

1. The problems of applying Hausdorff distance directly to trajectory data are high computational cost as it visits every pairwise sample points in two trajectories, and that it cannot distinguish the direction while computing because the distance between two trajectories is defined as the distance between two sample points from the corresponding trajectories under a certain criterion. In [10], Voronoi diagram is used to speedup the calculation of Hausdorff distance, but it is complicated to implement. On the other hand, the direction attribute can be added when computing distance, but the extension of feature will increase the computational cost. To solve this, a modified distance measure based on directed Hausdorff distance is proposed to calculate the difference between trajectories. In addition, the modified measure has the advantage of a fast computation, which meets the requirement of performing online learning in a fast manner.
2. According to the description in [10], when the data size is quite small, the new coming trajectory can be regarded to be abnormal, however, with time evolving, this trajectory may have enough similar neighbors to be identified as normal. Our solution is introducing a re-do step into the detection procedure to identify anomalous data more accurately.
3. The anomaly threshold is a critical parameter since it controls the sensitivity to true anomalies and error rate. As aforementioned, in [10], the threshold is manually selected which relies on the user experience. Instead of predefining the anomaly threshold, an adaptive and data-based method is proposed to make the algorithm more parameter-light, which is more easily applicable for practical use.

In addition, compared with the work in [10], this paper expands the experiments in two aspects:

1. In order to evaluate the performance of anomaly detection, F1-score is used in [10] to compare SHNN-CAD with different approaches. We propose to apply more performance measures, such as, precision, recall, accuracy, and false alarm rate, in order to analyze the behaviour of anomaly detection algorithms comprehensively.
2. One important advantage of Hausdorff distance is that it can deal with trajectory data with different number of sample points. However, in the experiments of evaluating SHNN-CAD [10], all the testing data have the same number of sample points. In this paper, the experiments are enriched by introducing more datasets with unequal length.

The rest of this paper is organized as follows. Following this introduction, Section 2 reviews unsupervised anomaly detection approaches of trajectory data. Section 3 gives a brief description of SHNN-CAD and interprets where SHNN-CAD can be improved for practical use, and then explains the SHNN-CAD${}^{+}$ that improves some limitations of SHNN-CAD. Section 4 presents extensive experiments on both real and synthetic datasets and discusses the performance of the proposed improvement strategies. Finally, concluding remarks and future work are given in Section 5.

## 2. Related Work

In the last few years, a branch of research have made effort to find efficient ways to detect outliers in trajectory data [6,11,12]. Since in reality, the available trajectories are usually raw data without any prior knowledge, this section focuses on the unsupervised approaches of anomaly detection which can be grouped into two categories: Clustering-based and non-clustering-based. Table 1 gives an overview of the approaches that are discussed below.

For clustering-based approaches, all the trajectories are clustered to obtain patterns, and the outliers are detected along with or after the clustering procedure due to the big difference with learnt patterns. The density-based spatial clustering of applications with noise (DBSCAN) algorithm [4,13] is of particular interest for both clustering and anomaly detection, considering that it is capable of discovering arbitrary shapes of clusters along with reporting outliers. However, the selection of two essential parameters limits its broad applicability. In concrete, DBSCAN requires two parameters, Eps and MinPts, to control the similarity between trajectories and the density of a cluster. The first one suffers from determining a proper distance measure which is still an open challenge [26]. The last one fails to support a good result when the densities of different clusters vary a lot. In addition, without the prior information of data, it is not straightforward and difficult to predefine specific parameters. Birant and Kut [14] proposed the Spatial-temporal DBSCAN (ST-DBSCAN) to improve DBSCAN by additionally dealing with the time attribute, but it increases the computational cost to calculate the similarity on both spatial and temporal dimension. It is well known that density based algorithms face with the problem of varied densities in data, Zhu et al. [15] proposed to solve this issue by developing a multi-dimensional scaling (DScale) method to readjust the computed distance.

Kumar et al. [16] proposed iVAT+ and clusiVAT+ for trajectory analysis along with detecting outliers. These approaches group the trajectories into different clusters by partitioning the Minimum Spanning Tree (MST). To build MST, the nondirectional dynamic time warping (DTW) distance between trajectories is regarded as the weight of the corresponding edge. The clusters which have very few trajectories are taken as irregular patterns, as a result the included trajectories are outliers. Obviously, the user expectation is necessary for determining how “few” should be.

Given the clusters, the testing trajectory is marked anomalous if the difference between it and the closest cluster center (also called centroid or medoid) is over an anomaly threshold. The representative distance measures that have been developed and applied in different applications are Euclidean distance (ED), Hausdorff distance, dynamic time warping (DTW), longest common subsequence (LCSS), etc. [26]. As the threshold is indirect to determine for varied practical situations, some approaches based on the probabilistic models have been proposed, and the distance is usually taken as the trajectory likelihood. In [17], the kernel density estimation (KDE) is used to detect the incoming sample point of the aircraft trajectory in progress. The sample point is determined as abnormal or belonging to a certain cluster depending on the probability is small or not. Guo et al. [18] proposed to apply the Shannon entropy to adaptively identify if the testing trajectory is normal or not. The normalized distances between the testing trajectory and the cluster centers obtained by the Information Bottleneck (IB) method build the probability distribution to compute the Shannon entropy, which measures the information used to detect the abnormality.

Some approaches attempt to detect outliers without the clustering procedure. In 2005, Keogh et al. [19] introduced the definition of time series discord and proposed the HOT Symbolic Aggregate ApproXimation (SAX) algorithm for the purpose of finding the subsequence (defined as discord) in a time series that is most different to all the rest subsequences. The authors proposed to search the discord via comparing the distance of each possible subsequence to the nearest non-self match using the brute force algorithm. Although the brute force algorithm is intuitive and simple, the time complexity is very high, which drives them to improve the process in a heuristic way. This definition was then improved and applied in different kind of time series including trajectory data by Yankov et al. [20] by treating each trajectory as a candidate subsequence.

Lee et al. [21] presented an efficient partition and detection framework, and developed a trajectory outlier detection algorithm TRAOD. Each trajectory is partitioned to a set of un-overlapping line segments based on the minimum description length (MDL) principle. Then the outlying line segments of a trajectory are picked. Due to the distance measure applied, the approach is able to detect both the positional and angular outliers. The novelty is that this algorithm is able to detect the outlying line segment other than the whole trajectory.

In [22], Guo et al. proposed a group-based signal filtering approach to do trajectory abstraction, where the outliers are filtered in an iterative procedure. Unlike the clustering algorithms, every trajectory may works in more than one group, and in the first phase (matching) the trajectory that has few similar items is considered as an outlier. In addition, the approach further picks the outliers in the second phase (detecting). It applies the 3 dimensional probability distribution function to represent the trajectory data and then computes Shannon entropy for outlier detection.

Banerjee et al. [23] designed the Maximal ANomalous sub-TRAjectories (MANTRA) to solve the problem of mining maximal temporally anomalous sub-trajectories in the field of road network management. The type of trajectory data studied is more specific and is called network-constrained trajectory which is a connected path in the road network. Thus, it is not easy to be applied to other anomaly detection applications.

Kanarachos et al. [25] proposed a systematic algorithm combining wavelets, neural networks and Hilbert transform for anomaly detection in time series. Being parameter-less makes the algorithm applicable in real world scenarios, for example, the anomaly threshold is given through the receiver operating characteristics without any assumption of data distribution.

Yuan et al. [24] tackled specific abnormal events for reminding drivers of danger. Both the location and direction of the moving object are taken into account, and contribute to the sparse reconstruction framework and the motion descriptor by a Bayesian model, respectively. Instead of dealing with raw trajectory data, this work is based on the video data where the object motion (trajectory) is represented by the pixel change between frames.

## 3. SHNN-CAD${}^{+}$: An Improvement of SHNN-CAD

First, the Sequential Hausdorff Nearest-Neighbor Conformal Anomaly Detector (SHNN-CAD) [10] is briefly described. Afterwards, we discuss several factors that influence the performance. Finally, we introduce the SHNN-CAD${}^{+}$.

### 3.1. SHNN-CAD Based Anomaly Detection

Laxhammar and Falkman proposed to perform online anomaly detection based on the conformal prediction [27] which estimates the *p*-value of each given label for a new observation, utilizing the non-conformity measure (NCM) to quantify the difference with the known observations. Successively three similar algorithms have been introduced [8,9,10] where SHNN-CAD is the last and the most complete one.

Considering that the conformal predictor provides valid prediction performance at arbitrary confidence level, Laxhammar and Falkman firstly defined the conformal anomaly detector (CAD). In concrete, given a training set $T=\left\{x_{1},x_{2},\ldots ,x_{l}\right\}$, a specified NCM, and a pre-set anomaly threshold ϵ, the corresponding nonconformity scores $\left(\alpha_{1},\alpha_{2},\ldots ,\alpha_{l+1}\right)$ are first computed. Then the *p*-value of $x_{l+1}$, $p_{l+1}$, is determined as the ratio of the number of trajectories that have greater or equal nonconformity scores to $x_{l+1}$ to the total number of trajectories.

(1) $$p_{l+1}=\frac{\left|\left\{\alpha_{i}|\alpha_{i}\geq \alpha_{l+1},1\leq i\leq l+1\right\}\right|}{l+1},$$

where $\left|\left\{.\right\}\right|$ computes the number of elements in the set. If $p_{l+1}<\epsilon$, then $x_{l+1}$ is identified as conformal anomaly, otherwise, $x_{l+1}$ is grouped to the normal set. Clearly, NCM is an essential factor that influences the quality of anomaly detection. The authors applied the k-nearest neighbors (kNN) and directed Hausdorff distance (DHD) to construct NCM, which refers to the Similarity based Nearest Neighbour Conformal Anomaly Detector (SNN-CAD) [9]. That is, the sum of distances between an observation $x_{i}$ and its k nearest neighbors, NNeighbor, is defined as the nonconformity score of this observation. Thus, the nonconformity score of $x_{i}$ is given by

(2) $$\alpha_{i}=\sum_{x_{j}\in NNeighbor}{\vec{d}}_{h}\left(x_{i},x_{j}\right),$$

where ${\vec{d}}_{h}\left(.\right)$ measures the distance between observations. In addition, since the new observation can be a single sample point, a line segment or a full trajectory, SNN-CAD was re-introduced as SHNN-CAD.

Usually unsupervised algorithms do not use any training set, and the outliers are defined to be observations which are far more infrequent than normal patterns [28]. In this paper we also adopt the “training set” concept as used in [10], assuming that the training set has only normal instances. In practical applications, due to the advantage of SHNN-CAD that only a small volume of data is needed as training set, these data can be chosen by users to make the algorithm work more effectively.

### 3.2. Discussion of SHNN-CAD

SHNN-CAD is not capable enough to adaptively detect outliers efficiently. The reason is threefold which is interpreted below.

First, using directed Hausdorff distance (DHD) to quantify the distance between trajectories cannot distinguish the difference of direction. DHD has the advantage of dealing with trajectory data with different number of sample points, showing the ability of computing distance for a single sample point or a line segment, which is important for the sequential anomaly detection of SHNN-CAD. Nonetheless, DHD was originally designed for point sets with no order between points as shown from the definition. Given two point sets, $A=\left\{a_{1},a_{2},\ldots ,a_{m}\right\}$ and $B=\left\{b_{1},b_{2},\ldots ,b_{n}\right\}$, the DHD from *A* to *B* is defined as

(3) $${\vec{d}}_{h}\left(A,B\right)=\max_{i=1}^{m}\left(\min_{j=1}^{n}d_{p}\left(a_{i},b_{j}\right)\right)$$

where *m* and *n* are the number of points in sets *A* and *B* (without loss of granularity, assuming that m≥n henceforth), respectively. $d_{p}\left(a_{i},b_{j}\right)$ returns the distance between points $a_{i}$ and $b_{j}$, which is usually obtained by Euclidean distance [29]. Computing the DHD matrix of data is the most time-consuming part of SHNN-CAD, while the time complexity of DHD, $O\left(mn\right)$, is high in the traditional computation way that visits every two sample points from corresponding trajectories, which is almost impractical for large size datasets in real world. Alternatively, in [10], the authors adopted the algorithm proposed by Alt [30] which benefits from Voronoi diagram to reduce the time complexity to $O\left(\left(m+n\right)\log\left(m+n\right)\right)$, but this algorithm requires to pre-process each trajectory by representing the included sample point based on its former neighbor. Moreover, although the trajectory is recorded as a collection of sample points, the order between points must be considered since it refers to the moving direction. For example, a car running in the lane with inverse direction should be detected as abnormal. Obviously, DHD is not sensitive for the direction attribute. As suggested in some literatures, the direction attribute at each sample point can be extracted and added to the feature matrix to obtain the distance, but the extra feature will increase the computational cost of distance measure. On the other hand, the direction attribute is generally computed as the intersection angle between line segment and horizontal axis, which may introduce noise to the feature matrix.

**Figure 1:**
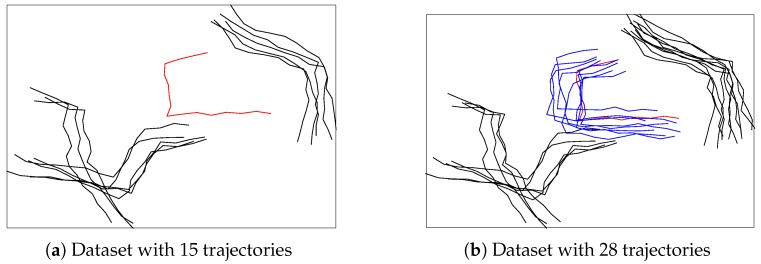
Plots of the trajectory change in dataset. As time goes on, from (**a**) to (**b**), the number of trajectories increases, and the red trajectory has several similar items (in blue color).

Second, SHNN-CAD is designed for online learning and anomaly detection which is highly desirable in practical applications, such as video surveillance. When a new observation is added into the database, SHNN-CAD decides it to be abnormal or not based on its *p*-value. As it can be seen in Equation (1), the *p*-value of an observation counts the amount of trajectories from the training set that have greater or equal nonconformity score to it. Obviously, the greater the *p*-value, the closer the observation to its *k* nearest neighbors, thus, it has higher probability of being normal. According to the mechanism of SHNN-CAD, once the trajectory comes to the dataset, it is identified as normal or not, and then the training set is updated for next testing trajectory. As shown in Figure 1a, the red trajectory has no similar items, thus it is detected as abnormal assuming that the *p*-value is bigger than the predefined anomaly threshold. However, unlike the case of a fixed dataset, the neighbors of an observation in online analysis are dynamic. In Figure 1b, the red trajectory has several similar items (in blue color), which means that its *p*-value may change to be greater than the anomaly threshold, and should be considered as normal. In conclusion, ignoring the influence of time evolution may bring errors in online anomaly detection.

Third, SHNN-CAD has two settings of anomaly threshold. The first one, $\frac{1}{l+1}$, is to deal with the problem of zero sensitivity when the dataset size, *l*, is small. The second one is a predefined ϵ ($\epsilon >\frac{1}{l+1}$). In the first case, zero sensitivity means that, as long as l<ϵ, an actually normal observation that has the smallest *p*-value, $\frac{1}{l+1}$, is identified as abnormal if there is only one threshold ϵ. Essentially, the first threshold defines the new coming observation as abnormal in default if it has the smallest *p*-value, $\frac{1}{l+1}$. However, this strategy causes another problem of zero precision of anomaly detection. For example, an actually abnormal observation that has the smallest *p*-value, is classified as normal. In the second case, in theory, when the anomaly threshold is equal to the prior unknown probability of abnormal class λ, SHNN-CAD achieves perfect performance. However, for unsupervised CAD, no background information can help to tune ϵ.

### 3.3. SHNN-CAD${}^{+}$

According to the previous discussion, three improvement strategies to enhance the performance of anomaly detection are proposed and explained clearly and thoroughly. Finally, the pseudocode of the SHNN-CAD${}^{+}$ method to detect a new coming is given and described.

First, as aforementioned, directly utilizing directed Hausdorff distance (DHD) for trajectory distance measure happens the problems of direction neglect and high computational complexity. The first problem is due to that DHD considers the two trajectories as point sets, which ignores the order between sample points, leading to the decline of accuracy. The second one is because that all the pairwise distances between the points of both sets have to be computed. To address the above issues, we propose to modify DHD by introducing a constraint window. The definition of DHD with constraint window, DHD(ω), from trajectory A to trajectory B is

(4) $${\vec{d}}_{hw}\left(A,B\right)=\left\{\begin{array}{cc} \max\left\{\max_{i=1}^{n+\omega }\left(\min_{|j-i|\leq w}d_{p}\left(a_{i},b_{j}\right)\right)\right\}, & m-n\leq w\\ \max\left\{\max_{i=1}^{n+\omega }\left(\min_{|j-i|\leq w}d_{p}\left(a_{i},b_{j}\right)\right),\max_{i=n+\omega +1}^{m}d_{p}\left(a_{i},b_{n}\right)\right\}, & m-n>w \end{array}\right.$$

where ω denotes the size of constraint window. Considering that unequal-length trajectories have a large difference in speed, ω is set as $\left\lceil \frac{m}{n}\right\rceil$ in this paper to limit that similar trajectories are homogeneous in speed. Obviously, each sample point in trajectory *A* visits at most 2ω+1 sample points in trajectory *B*, resulting in a linear time complexity $O\left(\left(m+n\right)\omega \right)$, where ω≪m,n. On the other hand, the search space is limited to the sample points that follow temporal order, which not only enables the consideration of direction, but also improves the accuracy for measuring the distance as an extended visit may introduce wrong matching between two trajectories. Note that the direction of a sample point is an important attribute to give higher performance of detecting abnormal events for trajectory data in practical applications, such as vehicle reverse driving.

**Figure 2:**
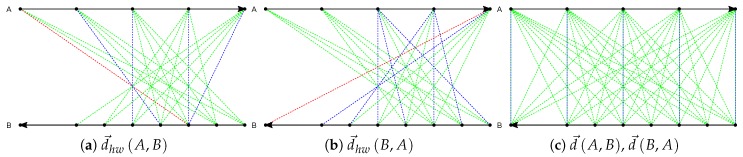
Plots of the distance between two trajectories A and B by directed Hausdorff distance with window (DHD(ω)) and directed Hausdorff distance (DHD). (**a**) distance from A to B by DHD(ω). (**b**) distance from B to A by DHD(ω). (**c**) distance from A to B and from B to A by DHD.

Figure 2 gives an example to illustrate the distance computation. Trajectories A and B have 5 and 8 sample points, respectively, and they have opposite directions which are indicated by the arrows at the end points. The dashed lines visualized in red, green, and blue colors represent the distances between sample points that are required to compute the distance two trajectories. The shortest distance between one sample point and its corresponding points from another trajectory is shown in blue or red. The red line indicates the maximum from all the shortest distances, namely the distance between A and B. Suppose that the window size is 2, the distances from A to B and from B to A by DHD(ω) are computed as illustrated in Figure 2a and Figure 2b, respectively. Figure 2c shows the distance computation by DHD. The distances from A to B and from B to A are the same (all the blue lines are equal). In contrast, DHD(ω) saves the computational cost. Additionally, due to that the order of sample points is taken into account, DHD(ω) captures the difference between trajectories more accurately than DHD with respect to different features in trajectory data.

Second, considering that in online learning, the outlier may turn into normal once it has enough similar trajectories, we propose to apply a re-do step. To save time, not all outliers are rechecked again with every new coming. According to the theory of conformal anomaly detector, the anomaly threshold ϵ indicates how much probability of outliers occur in the dataset. Thus, if the size of outliers arrives larger than expected, several previous detected outliers may be detected as normal. For example, when the size of training data is *l*, if the new coming $x_{l+1}$ is identified as outlier but the number of outliers is greater than expected $\left(l+1\right)\cdot \epsilon$, the previous outliers will be rechecked if they can be moved to the normal class or not. In particular, this strategy helps to solve the problem of zero precision by SHNN-CAD when the data size is small. Regarding the use of predefined anomaly threshold, it is more reasonable to treat the new coming as abnormal when its p-value is too small, which means it has few similar items from the training set. Via the re-do strategy, the outliers can be picked out gradually with new comings. We don’t recheck the normal ones again, because their similar trajectories may increase or remain the same. In fact, for large volume data, the normal trajectories are usually pruned to discard redundant information or are trained into mixtures of models to avoid high computational cost, which will be considered in the future work.

Third, automated identification of outliers requires data-adaptive anomaly threshold instead of explicitly adjusting for different kind of trajectory datasets. Unlike SHNN-CAD, we define the threshold for the new coming when the size of training set is *l* as the minimum probability of a normal trajectory from training set $N_{l}$.

(5) $$\epsilon_{l}=\min_{i\in N_{l}}p_{i}$$

where $p_{i}$ is the *p*-value of *i*th trajectory. This setting is intuitive and straightforward since the *p*-values of the other trajectories in the training set are greater or equal to the defined anomaly threshold, which enables them to be normal. Obviously, the determination of ϵ depends on the condition of the considering training set, which makes the approach more applicable for different datasets.

Algorithm 1 shows how SHNN-CAD${}^{+}$ works for a new coming. Compared with SHNN-CAD, the previously detected outliers are also input to perform the re-do strategy. Given the input, two zero distance arrays are built for the new coming $x_{l+m+1}$ (Lines 1 and 2). Lines 3–10 compute the nonconformity scores of all trajectories by summing the distances with the *k* nearest neighbors. The element $D_{i,j}$ denotes the distance from *i*th trajectory to its *j*th neighbor, which is obtained through modified Hausdorff distance. Then the *p*-values are calculated in Lines 11–12 according to Equation (1). Differing from SHNN-CAD, the anomaly threshold ϵ is dynamically updated depending on the training set (Line 13). From Line 14 to 25, the new coming is identified as outlier or not with the defined ϵ, and the outlier and training sets are updated correspondingly. If the size of the outlier set is over the expected value, the re-do strategy is performed on each previous outlier to check if it can be turned to the normal set (Lines 17–22).

**Algorithm 1:** Adaptive Online Trajectory Anomaly Detection with SHNN-CAD${}^{+}$

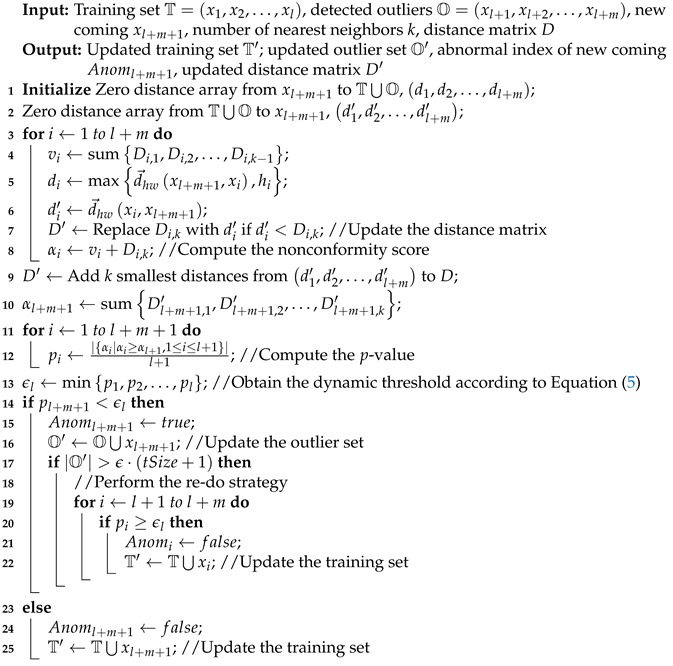



## 4. Experiments

In this section, we present the experiments conducted. The matlab implementation is available at [31]. Firstly, a comparison of the proposed DHD$\left(\omega \right)$ to the typical DHD is given. Secondly, the performance of applying DHD$\left(\omega \right)$ to the anomaly detection measure is analyzed. Finally, the improvement on SHNN-CAD is evaluated. All the experimental results in this paper are obtained by MATLAB 2018a software running on a Windows machine with Intel Core i7 2.40 GHZ CPU and 8 GB RAM.

### 4.1. Comparison of Distance Measure

To evaluate the performance of measuring distance of DHD$\left(\omega \right)$, we adopt the 10 cross-validation test using 1-Nearest Neighbor (1NN) classifier which has been demonstrated to work well to achieve this goal [26,32]. 1NN is parameter-free and the classification error ratio of 1NN only depends on the performance of the distance measure. Initially, the dataset is randomly divided into 10 groups. Then each group is successively taken as testing set, and the rest work as the training set for 1NN classifier. Finally, each testing trajectory is classified into the same cluster with its nearest neighbor in the training set. The 10 cross-validation test runs 100 times to obtain average error ratio. The classification error ratio of each run is calculated as follows:

(6) $$\mathrm{classification}\;\mathrm{error}\;\mathrm{ratio}=\frac{1}{10}\sum_{i=1}^{10}\frac{\mathrm{number}\;\mathrm{of}\;\mathrm{trajectories}\;\mathrm{wrongly}\;\mathrm{classified}}{\mathrm{number}\;\mathrm{of}\;\mathrm{trajectories}\;\mathrm{in}\;\mathrm{the}\;i\mathrm{th}\;\mathrm{testing}\;\mathrm{set}}$$

**Figure 3:**
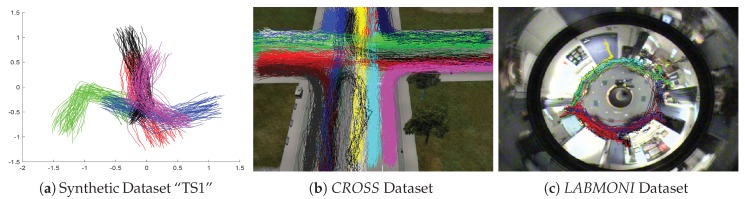
Plots of trajectory datasets used for the evaluation of distance measures. Trajectories in the same cluster have the same color.

One thousand synthetic, 1 simulated, and 1 real trajectory datasets are utilized in this experiment. The *Synthetic Trajectories I* (numbered “I” to distinguish from the datasets in Section 4.3) is generated by Piciarelli et al. [33], which includes 1000 datasets. In each dataset, 250 trajectories are equally divided into 5 clusters and the remaining 10 trajectories are abnormal (abnormal trajectories are not considered in this experiment), see an example in Figure 3a. Each trajectory is recorded by the locations of 16 sample points. The simulated dataset *CROSS* and real dataset *LABOMNI* are contributed by Morris and Trivedi [34,35]. The trajectories in *CROSS* are designed to happen in a four way traffic intersection as shown in Figure 3b. *CROSS* includes 1999 trajectories which evenly belong to 19 clusters, and the number of sample points varies from 5 to 23. The trajectories in *LABOMNI* are from humans walking through a lab as shown in Figure 3c. *LABOMNI* has 209 trajectories from 15 clusters and the number of sample points varies from 30 to 624.

Table 2 gives the comparison results of the two different distance measures in terms of the average and standard deviation (std) of the classification error ratio. Due to the limited space of this paper, only the average result of the 1000 datasets in *Synthetic Trajectories I* is given. From the results on all datasets, we can see that DHD(ω) works better than DHD to measure the difference between trajectories with only having the location information. In particular, in the case of real dataset *LABOMNI*, the classification performance improves a lot with the use of DHD(ω), which demonstrates that DHD(ω) captures the difference between trajectories more accurately than DHD. Compared with the datasets of *Synthetic Trajectories I* and CROSS, the trajectories in *LABOMNI* are more complex in twofold: First, the number of sample points varies more dramatically; second, the trajectories with opposite directions are more close in location, for example, the trajectories following the same traffic rules in CROSS are distributed in different lanes. In the final column of Table 2, the *p*-value for the null hypothesis that the results from the two distance measures are similar by Kruskal-Wallis test [36] is presented. The results of *Synthetic Trajectories I* and *LABOMNI* are largely different at a 1% significance level, while the difference in CROSS is not so great. Note that *Synthetic Trajectories I* includes 1000 datasets, thus in conclusion, the results between DHD(ω) and DHD are significantly different.

In addition, we compare the distance measures on time series data which is more general since the trajectory data is a specific form of time series [37]. The results are consistent with the expectations as shown in Table A1, Appendix A.

### 4.2. Comparison of Anomaly Detection Measures

The directed Hausdorff k-nearest neighbors nonconformity (DH-kNN NCM) measure is the core part of SHNN-CAD which computes the nonconformity score and further contributes to calculating the *p*-value of testing trajectory for classification. In this section, we evaluate the performance of DH-kNN NCM with the utilization of DHD(ω). The same datasets and criterion used in [10] are adopted for comparative analysis, in addition, a real dataset from [18] is tested.

**Figure 4:**
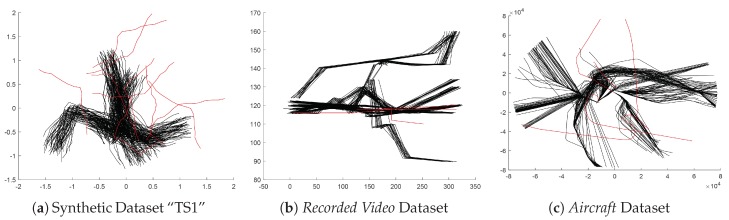
Plots of trajectory datasets used for the evaluation of anomaly detection measures. The trajectories colored with red are abnormal. (**a**) One dataset “TS1” from synthetic datasets. (**b**) *Recorded Video* trajectory Dataset. (**c**) *Aircraft* trajectory Dataset.

The 1000 synthetic trajectory datasets mentioned in Section 4.1 are the first group of testing data. Note that the outliers in each dataset are also included in the experiment, see Figure 4a. The second dataset consisting of 238 recorded video trajectories is provided by Lazarević et al. [38]. Each trajectory includes 5 sample points and is labeled as normal or not. In this dataset, only 2 trajectories are abnormal, as shown in Figure 4b. The *Aircraft* Dataset used by Guo et al. includes 325 aircraft trajectories with the number of sample points varying between 102 and 1023, and 5 trajectories are labeled as abnormal [18], as shown in Figure 4c. For each dataset, the nonconformity scores of all the trajectories are calculated and sorted. The accuracy of anomaly detection is calculated as the proportion of outliers in the top *n* nonconformity scores. Here, *n* is the number of outliers in the dataset according to the groundtruth.

Table 3 shows the accuracy performances of DH-kNN NCM and the version with DHD(ω) on 1002 datasets. Due to the space limit of this paper, only the average result of the 1000 datasets in *Synthetic Trajectories I* is given. For *Synthetic Trajectories I*, using DHD(ω) improves the detection quality of DH-kNN NCM regardless of the number of nearest neighbors *k*. Additionally, with DHD and DHD(ω), the anomaly detection measure works the best when k=2. For *Recorded Video Trajectories* and *Aircraft Trajectories*, the replacement of DHD(ω) achieves the same detection result.

### 4.3. Comparison of Online Anomaly Detection

In order to demonstrate the efficiency and reliability of the proposed improvement, we compare the performance of SHNN-CAD${}^{+}$ with SHNN-CAD on the same datasets applied in [10] and further introduce more datasets.

The synthetic trajectories [39] presented in [10] for online anomaly detection is created by Laxhammar using the trajectory generator software written by Piciarelli et al. [40]. *Synthetic Trajectories II* includes 100 datasets, and each dataset has 2000 trajectories. Each trajectory has 16 sample points recorded with location attribute and has the probability 1% of being abnormal. To expand the dataset for experiment, we reuse *Synthetic Trajectories I* and rename it by *Synthetic Trajectories III* [41]. The trajectories in each dataset of *Synthetic Trajectories I* are randomly reordered since they are organized regularly by cluster, which is not common in practical applications. In addition, considering Hausdorff distance has the advantage of dealing with trajectory data with different number of sample points, however, in the experiments of [10], all the testing data have equal length for online learning and anomaly detection, we produce a collection of datasets where the trajectories have various lengths, called *Synthetic Trajectories IV* [41]. The trajectory generator software [40] is enhanced to produce trajectories with the number of sample points ranging from 20 to 100. For each dataset, firstly, 2000 normal trajectories from 10 equal-size clusters are generated with the randomness parameter 0.7 and are reordered to simulate the real scene. Then 1000 abnormal trajectories are generated. Finally, each normal trajectory is independently replaced with the probability λ by an abnormal one. The collection has 3 groups of datasets with λ equal to 0.005, 0.01, and 0.02, respectively, and each group contains 100 trajectory datasets.

In [10], *F*1-score is utilized to compare the overall performance of online learning and anomaly detection. *F*1-score is the harmonic mean of precision and recall (also called detection rate in the field of anomaly detection). In addition to this, we also analyze the false alarm rate and accuracy values for the purpose of comprehensive evaluation from different aspects [42]. Precision indicates the proportion of true outliers in the detected abnormal set. Recall presents the percentage of outliers that are detected. Accuracy computes ratio of correctly classified (normal or abnormal) trajectories. False alarm rate measures the rate of wrongly detecting an outlier. The calculations of these performance measures are as follows:

(7) $$\mathrm{precision}=\frac{\mathrm{number}\;\mathrm{of}\;\mathrm{anomalies}\;\mathrm{detected}}{\mathrm{number}\;\mathrm{of}\;\mathrm{objects}\;\mathrm{classified}\;\mathrm{as}\;\mathrm{anomalies}}$$

(8) $$\mathrm{recall}\;\left(\mathrm{detection}\;\mathrm{rate}\right)=\frac{\mathrm{number}\;\mathrm{of}\;\mathrm{anomalies}\;\mathrm{detected}}{\mathrm{total}\;\mathrm{number}\;\mathrm{of}\;\mathrm{anomalies}\;\mathrm{labeled}\;\mathrm{in}\;\mathrm{groundtruth}}$$

(9) $$F1=\frac{2\cdot \mathrm{precision}\cdot \mathrm{recall}}{\mathrm{precision}+\mathrm{recall}}$$

(10) $$\mathrm{accuracy}=\frac{\mathrm{number}\;\mathrm{of}\;\mathrm{trajectories}\;\mathrm{correctly}\;\mathrm{classified}}{\mathrm{total}\;\mathrm{number}\;\mathrm{of}\;\mathrm{trajectories}}$$

(11) $$\mathrm{false}\;\mathrm{alarm}\;\mathrm{rate}=\frac{\mathrm{number}\;\mathrm{of}\;\mathrm{normal}\;\mathrm{trajectories}\;\mathrm{classified}\;\mathrm{as}\;\mathrm{abnormal}}{\mathrm{total}\;\mathrm{number}\;\mathrm{of}\;\mathrm{normal}\;\mathrm{trajectories}\;\mathrm{labeled}\;\mathrm{in}\;\mathrm{groundtruth}}$$

Firstly, the performance of SHNN-CAD and SHNN-CAD${}^{+}$ is compared using the aforementioned 1400 trajectory datasets. *k* is set to 2 as suggested in [10]. The average results for each collection of trajectory data are given in Table 4 where the best score of each performance measure is highlighted. Note that it is necessary to pre-set the anomaly threshold ϵ for SHNN-CAD, thus, we define ϵ based on the real probability of anomaly λ. For *Synthetic Trajectories III*, the λ is computed as 10/260 as each dataset has 10 outliers and 250 normal trajectories. It is clear from the table that SHNN-CAD works the best in most datasets with regard to the F1 score when ϵ is close to λ, which is consistent with expected and with the description in [10]. Compared to the results in this case, SHNN-CAD${}^{+}$ achieves better results. It is important to point out that for unsupervised anomaly detection, λ is not available and no information can be used to help to determine ϵ. For example, in different collection of datasets, ϵ is set with a different value. In addition, SHNN-CAD${}^{+}$ achieves better results in all the datasets with the accuracy index. Thus, SHNN-CAD is less applicable in real-world applications than SHNN-CAD${}^{+}$, which utilizes the adaptive anomaly threshold. Furthermore, the average running time of dealing with 2000 equal-length trajectories in *Synthetic Trajectories II* is 39.85 s by SHNN-CAD${}^{+}$. For comparison, the typical implementation of DHD with computing distance between every pairwise sample points is equipped to the anomaly detection procedure of SHNN-CAD to get the running time, which is 128.08 s in this case. For unequal-length 2000 trajectories in *Synthetic Trajectories IV*, the running time becomes longer as 109.34 s by SHNN-CAD${}^{+}$ and 556.28 s by SHNN-CAD with typical DHD implementation. Note that optimal implementations (as the one suggested in [10], which is based on the Voronoi diagram) will improve the result.

Next, in order to test the relative performance of each proposed improvement strategies, we conduct experiments based on three objectives. First (Objective 1), to verify that DHD(ω) helps to improve the performance of SHNN-CAD, SHNN-CAD is implemented with DHD(ω) computing the distance between trajectories. Second (Objective 2), for the purpose of demonstrating the rationality of the re-do step, SHNN-CAD is equipped with a re-do step in the procedure of anomaly detection. Third (Objective 3), to prove the effectiveness of adaptive anomaly threshold, the predefinition of ϵ is replaced in SHNN-CAD. The results are listed in Table 5. For the task of objective 1 and objective 2, the results are compared with those by SHNN-CAD in Table 4. In the case of objective 1, obviously, the utilization of DHD(ω) improves the behaviour of anomaly detection regardless of most performance measures for all the datasets. In the case of objective 2, only the recall index for some datasets is not as well as SHNN-CAD, which means the missing recognition of outliers. However, the comprehensive F1 score indicates that the performance is promising. In the case of objective 3, the results are compared with SHNN-CAD in Table 4 when ϵ is closest to the corresponding λ. Clearly, for most datasets, the adaptive anomaly threshold can make up the shortcoming of predefinition and strengthen the capability of anomaly detection. Compared with the SHNN-CAD${}^{+}$ in Table 4, all the improvement strategies work together to accomplish the enhancement of SHNN-CAD.

## 5. Conclusions

Based on the Sequential Hausdorff Nearest-Neighbor Conformal Anomaly Detector (SHNN-CAD), which focuses on online detecting outliers from trajectory data, we have presented an enhanced version, called SHNN-CAD${}^{+}$, to improve the anomaly detection performance. The proposal includes three improvement strategies: First, modifying typical point-based Hausdorff distance to be suitable for trajectory data and to be faster in distance calculation; second, adding a re-do step to avoid false positives in the initial stages of the algorithm; third, defining data-adaptive and dynamic anomaly threshold rather than a pre-set and fixed one. Experimental results have shown that the performance of the presented approach has been improved over SHNN-CAD. Considering that the training set will increase a lot with time, further research will focus on incremental learning which prunes the historical data for future process.

## Figures and Tables

**Table 1:** Overview of anomaly detection in several related works.

Ref.	Category of Algorithm	Type of Data	Threshold	Evaluation Measure
[4]	clustering-based (DBSCAN)	trajectory data	implied	discuss with data managers
[13]	clustering-based (DBSCAN)	point set	implied	compare result with groundtruth, running time
[14]	clustering-based (ST-DBSCAN)	spatial-temporal data	implied	running time complexity, interpret results in application
[15]	clustering-based (DBSCAN/OPTICS/DP)	point set	implied	F-measure
[16]	clustering-based (iVAT+/clusiVAT+)	trajectory data	predefined	partition accuracy, false alarm, true positive
[17]	clustering-based (DBSCAN+KDE)	trajectory data	predefined	10-fold cross validation test, interpret results in application
[18]	clustering-based (IB+Shannon entropy)	trajectory data	automatic	accuracy, precision recall, F-measure
[19]	non-clustering-based (HOT SAX)	time series	automatic	interpret results with data, running time complexity
[20]	non-clustering-based (disk aware algorithm)	time series	automatic	running time
[21]	non-clustering-based (TRAOD)	trajectory data	predefined	pruning power, accuracy of pruning, speedup ratio
[22]	non-clustering-based (trajectory abstraction)	trajectory data	predefined	degree of redundancy, informativeness, precision, recall
[23]	non-clustering-based (MANTRA)	trajectory data	predefined	growth rate of running time/number of anomalous edges, accuracy, 5-fold cross validation, F-measure
[24]	non-clustering-based (anomaly detection in traffic scenes)	video data	automatic	pixel-wise receiver of characteristics (ROC), area under ROC
[25]	non-clustering-based (an algorithm combining wavelets, neural networks and Hilbert transform)	time series	automatic	false positive/alarm rate, true positive rate (hit rate), interpret results with data

**Table 2:** Classification Error Ratio (%) on Different Trajectory Datasets and the corresponding *p*-value.

	Distance Measures	DHD		DHD(ω)		*p*-Value
Datasets		Average	Std	Average	Std	
*Synthetic Trajectories I* (average)		0.1634	0.0032	0.1566	0.0031	≪0.001
*CROSS*		0.6100	0.0792	0.5937	0.0694	0.2182
*LABOMNI*		31.23	1.37	10.20	0.87	≪0.001

**Table 3:** Accuracy (%) of anomaly detection on different trajectory datasets.

Datasets	Nonconformity Measures	# of Most Similar Neighbors Considered				
		k=1	k=2	k=3	k=4	k=5
*Synthetic Trajectories I* (average)	DH-kNN NCM	96.42	97.09	97.05	96.95	96.77
	using DHD(ω)	96.45	97.85	97.81	97.74	97.65
*Recorded Video Trajectories*	DH-kNN NCM	100.00	100.00	100.00	100.00	100.00
	using DHD(ω)	100.00	100.00	100.00	100.00	100.00
*Aircraft Trajectories*	DH-kNN NCM	80.00	80.00	80.00	80.00	80.00
	using DHD(ω)	80.00	80.00	80.00	80.00	80.00

**Table 4:** Five Performance Measures (%) of Online Learning and Anomaly Detection on Different Trajectory Datasets. Note that we found a mistake in Table 3 of [10] where the given result of Sequential Hausdorff Nearest-Neighbor Conformal Anomaly Detector (SHNN-CAD) is different with the description of **Algorithm 2** in [10]. The F1 result, 53.52, 74.61, and 61.68, of SHNN-CAD with ϵ=0.005,0.01, and 0.02, respectively, is given under the condition that if *p*-value ≤ϵ, the testing trajectory is classified as abnormal. In the table below, we follow the rule in **Algorithm 2** [10] for SHNN-CAD. The best performance of each collection of datasets is in bold.

Trajectory Datasets	Approaches		Precision	Recall	F1	Accuracy	False Alarm Rate
*Synthetic Trajectories II* (λ=0.01)	SHNN-CAD	ϵ=0.005	**98.70**	40.39	54.75	99.39	**0.01**
		ϵ=0.01	87.15	77.48	79.80	99.63	0.13
		ϵ=0.02	50.24	**94.59**	64.35	98.98	0.98
	SHNN-CAD${}^{+}$		88.41	89.64	**86.38**	**99.77**	0.13
*Synthetic Trajectories III* (λ=0.038)	SHNN-CAD	ϵ=0.03	**97.34**	55.51	67.92	98.19	**0.10**
		ϵ=0.04	91.52	73.95	79.36	98.63	0.38
		ϵ=0.05	80.01	**83.40**	**79.83**	98.39	1.01
	SHNN-CAD${}^{+}$		84.75	82.70	79.39	**98.68**	0.70
*Synthetic Trajectories IV* (λ=0.005)	SHNN-CAD	ϵ=0.004	**90.97**	54.53	63.78	99.73	**0.03**
		ϵ=0.005	83.82	65.04	69.40	99.75	0.07
		ϵ=0.01	52.63	88.21	64.01	99.53	0.41
	SHNN-CAD${}^{+}$		78.43	**91.76**	**81.47**	**99.82**	0.15
*Synthetic Trajectories IV* (λ=0.01)	SHNN-CAD	ϵ=0.005	**99.17**	37.31	52.39	99.33	**0.00**
		ϵ=0.01	89.31	74.31	79.31	99.61	0.11
		ϵ=0.02	52.27	**92.09**	65.60	99.01	0.92
	SHNN-CAD${}^{+}$		88.64	89.52	**85.66**	**99.75**	0.14
*Synthetic Trajectories IV* (λ=0.02)	SHNN-CAD	ϵ=0.01	**98.99**	45.79	61.64	98.91	**0.01**
		ϵ=0.02	87.47	83.88	**84.62**	99.42	0.26
		ϵ=0.03	63.18	**93.02**	74.53	98.78	1.10
	SHNN-CAD${}^{+}$		95.36	78.75	81.45	**99.48**	0.10

**Table 5:** Five Performance Measures (%) of Proposed Improvement Strategies on Different Trajectory Datasets.

Trajectory Datasets	Approaches		Precision	Recall	F1	Accuracy	False Alarm Rate
*Synthetic Trajectories II* (λ=0.01)	Objective 1	ϵ=0.005	98.86	40.23	54.89	99.38	0.01
		ϵ=0.01	87.93	77.91	80.38	99.64	0.12
		ϵ=0.02	50.77	95.16	64.89	98.99	0.97
	Objective 2	ϵ=0.005	98.70	40.39	54.75	99.39	0.01
		ϵ=0.01	87.15	77.48	79.80	99.63	0.13
		ϵ=0.02	50.24	94.59	64.35	98.98	0.98
	Objective 3		87.08	87.21	84.97	99.75	0.13
*Synthetic Trajectories III* (λ=0.038)	Objective 1	ϵ=0.03	97.01	55.78	67.99	98.21	0.10
		ϵ=0.04	91.86	74.31	79.81	98.66	0.37
		ϵ=0.05	80.41	83.94	80.30	98.43	1.00
	Objective 2	ϵ=0.03	97.34	55.51	67.92	98.19	0.10
		ϵ=0.04	91.52	73.95	79.36	98.63	0.38
		ϵ=0.05	80.01	83.40	79.83	98.39	1.01
	Objective 3		85.10	82.25	79.02	98.64	0.72
Synthetic Trajectories IV (λ=0.005)	Objective 1	ϵ=0.004	93.88	58.22	67.45	99.75	0.02
		ϵ=0.005	87.23	69.39	73.28	99.78	0.06
		ϵ=0.01	52.75	91.08	65.01	99.54	0.41
	Objective 2	ϵ=0.004	90.97	54.53	63.78	99.73	0.03
		ϵ=0.005	83.82	65.04	69.40	99.75	0.07
		ϵ=0.01	52.63	88.21	64.01	99.53	0.41
	Objective 3		77.90	86.10	78.30	99.79	0.14
Synthetic Trajectories IV (λ=0.01)	Objective 1	ϵ=0.005	99.53	37.74	53.20	99.34	0.00
		ϵ=0.01	92.35	77.94	82.84	99.68	0.08
		ϵ=0.02	53.80	94.76	67.59	99.07	0.89
	Objective 2	ϵ=0.005	99.17	37.31	52.39	99.33	0.00
		ϵ=0.01	89.31	74.31	79.31	99.61	0.11
		ϵ=0.02	52.27	92.09	65.60	99.01	0.92
	Objective 3		87.55	84.08	82.63	99.69	0.14
Synthetic Trajectories IV (λ=0.02)	Objective 1	ϵ=0.01	99.60	45.80	61.70	98.92	0.01
		ϵ=0.02	90.18	86.59	87.38	99.52	0.20
		ϵ=0.03	65.68	95.28	77.02	98.91	1.02
	Objective 2	ϵ=0.01	98.99	45.79	61.64	98.91	0.01
		ϵ=0.02	87.47	83.88	84.62	99.42	0.26
		ϵ=0.03	63.18	93.02	74.53	98.78	1.10
	Objective 3		95.05	72.66	77.94	99.37	0.10

## Abbreviations

The following abbreviations are used in this manuscript:

HD	Hausdorff distance
DHD	Directed Hausdorff distance
DHD(ω)	Directed Hausdorff distance with constraint window
kNN	k-nearest neighbors
CAD	Conformal anomaly detector
NCM	Non-conformity measure
SNN-CAD	Similarity based Nearest Neighbour Conformal Anomaly Detector
SHNN-CAD	Sequential Hausdorff Nearest-Neighbor Conformal Anomaly Detector
SHNN-CAD${}^{+}$	Enhanced version of Sequential Hausdorff Nearest-Neighbor Conformal Anomaly Detector

## Appendix A. 10-Fold Cross Validation Results on 65 Time Series Datasets

As interpreted in [37], “A sequence composed by a series of nominal symbols from a particular alphabet is usually called a *temporal sequence* and a sequence of continuous, real-valued elements, is known as a *time series*”. According to the definition, trajectory data is a specific kind of time-series. We test the performance of DHD(ω) based on 65 public time series datasets which are collected by Chen et al. [43]. The average and stand deviation (Std) of classification error ratio on the datasets are given in Table A1 where DHD(ω) performs better than DHD.

**Table A1:** Classification Error Ratio (%) on Time Series Datasets. The best performance of each dataset is in bold.

No.	Dataset	Data Size	Clusters Size	DHD		DHD(ω)	
				Average	Std	Average	Std
1	50words	905	50	86.52	**0.0300**	**40.77**	0.0391
2	Adiac	781	37	71.33	0.0603	**36.48**	**0.0452**
3	ArrowHead	210	3	50.00	0.0985	**10.00**	**0.0613**
4	Beef	60	5	55.00	**0.2364**	**50.00**	0.2606
5	BeetleFly	40	2	40.00	**0.1748**	**22.50**	0.2486
6	BirdChicken	40	2	22.50	0.3217	**20.00**	**0.1581**
7	Car	120	4	58.33	0.1521	**29.17**	**0.0982**
8	CBF	930	3	60.54	0.0608	**3.44**	**0.0167**
9	Coffee	56	2	25.33	0.1501	**1.67**	**0.0527**
10	Computers	500	2	**26.40**	**0.0610**	37.80	0.0614
11	Cricket_X	780	12	78.08	**0.0293**	**48.33**	0.0897
12	Cricket_Y	780	12	80.51	0.0550	**50.51**	**0.0510**
13	Cricket_Z	780	12	78.33	**0.0333**	**48.08**	0.0397
14	DiatomSizeReduction	322	4	8.39	0.0392	**0.00**	**0.0000**
15	DistalPhalanxOutlineAgeGroup	539	3	33.19	0.0637	**23.38**	**0.0583**
16	DistalPhalanxOutlineCorrect	876	2	34.60	0.0509	**23.29**	**0.0425**
17	DistalPhalanxTW	539	6	43.21	0.0563	**29.68**	**0.0488**
18	Earthquakes	461	2	**32.34**	**0.0735**	36.04	0.0797
19	ECG200	200	2	31.50	0.1180	**11.50**	**0.0914**
20	ECG5000	5000	5	13.70	0.0173	**6.68**	**0.0114**
21	ECGFiveDays	884	2	3.51	0.0125	**0.00**	**0.0000**
22	FaceAll	2247	14	64.49	0.0278	**6.54**	**0.0149**
23	FaceFour	112	4	50.91	0.1621	**17.05**	**0.0911**
24	FacesUCR	2247	14	64.53	0.0337	**6.19**	**0.0135**
25	FISH	350	7	69.43	**0.0687**	**17.43**	0.0888
26	Gun_Point	200	2	39.50	0.1012	**2.00**	**0.0258**
27	Ham	214	2	45.28	0.0894	**27.62**	**0.0831**
28	Haptics	463	5	69.77	**0.0424**	**64.37**	0.0510
29	Herring	128	2	**45.45**	**0.1015**	46.79	0.1380
30	InsectWingbeatSound	2200	11	87.91	**0.0221**	**41.50**	0.0263
31	ItalyPowerDemand	1096	2	32.39	0.0485	**5.75**	**0.0206**
32	LargeKitchenAppliances	750	3	**51.33**	**0.0594**	60.53	0.0623
33	Lighting2	121	2	36.28	0.1149	**35.64**	**0.1137**
34	Lighting7	143	7	**52.43**	**0.0966**	54.48	0.1559
35	Meat	120	3	10.83	0.0883	**7.50**	**0.0730**
36	MedicalImages	1140	10	47.72	0.0310	**25.88**	**0.0262**
37	MiddlePhalanxOutlineAgeGroup	554	3	44.42	0.0928	**31.41**	**0.0662**
38	MiddlePhalanxOutlineCorrect	891	2	39.73	0.0648	**27.84**	**0.0480**
39	MiddlePhalanxTW	553	6	49.19	**0.0419**	**45.94**	0.0434
40	MoteStrain	1272	2	27.51	0.0384	**12.57**	**0.0285**
41	OliveOil	60	4	26.67	0.1610	**16.67**	**0.1361**
42	OSULeaf	442	6	65.16	**0.0443**	**38.71**	0.0926
43	PhalangesOutlinesCorrect	2658	2	37.77	0.0409	**24.08**	**0.0261**
44	Plane	210	7	21.90	0.0784	**2.86**	**0.0246**
45	ProximalPhalanxOutlineAgeGroup	605	3	29.40	**0.0554**	**23.64**	0.0620
46	ProximalPhalanxOutlineCorrect	891	2	30.18	**0.0493**	**18.30**	0.0541
47	ProximalPhalanxTW	605	6	33.88	**0.0560**	**26.42**	0.0824
48	RefrigerationDevices	750	3	**35.73**	**0.0439**	63.60	0.0507
49	ScreenType	750	3	**51.20**	**0.0467**	65.60	0.0474
50	ShapeletSim	200	2	**38.50**	0.1435	47.00	**0.0919**
51	ShapesAll	1198	60	83.48	**0.0225**	**21.37**	0.0302
52	SmallKitchenAppliances	750	3	**49.87**	0.0706	59.33	**0.0587**
53	SonyAIBORobotSurface	621	2	18.69	0.0346	**1.45**	**0.0119**
54	Strawberry	983	2	7.63	0.0183	**4.17**	**0.0170**
55	SwedishLeaf	1122	15	67.21	0.0525	**17.02**	**0.0359**
56	Symbols	1000	6	77.90	0.0370	**4.40**	**0.0222**
57	synthetic_control	600	6	73.50	0.0552	**3.33**	**0.0192**
58	ToeSegmentation1	252	2	30.51	**0.0697**	**26.09**	0.0912
59	ToeSegmentation2	166	2	**19.85**	**0.0784**	21.25	0.1409
60	Trace	200	4	25.00	0.0943	**6.50**	**0.0626**
61	TwoLeadECG	1162	2	8.86	0.0269	**0.95**	**0.011**
62	Wine	111	2	5.45	0.0878	**4.55**	**0.0643**
63	WordsSynonyms	905	25	82.75	**0.0458**	**38.12**	0.0489
64	Worms	225	5	**59.57**	**0.0839**	69.94	0.1074
65	WormsTwoClass	225	2	**40.45**	0.1212	50.61	**0.0490**
Average of all datasets				44.36	0.0744	**26.50**	**0.0640**
